# Proximity ligation *in situ *assay for monitoring the global DNA methylation in cells

**DOI:** 10.1186/1472-6750-11-31

**Published:** 2011-04-06

**Authors:** Eric Hervouet, Philippe Hulin, François M Vallette, Pierre-François Cartron

**Affiliations:** 1 Centre de Recherche en Cancérologie Nantes-Angers, INSERM, U892, Equipe Apoptose et Progression Tumorale, Equipe labellisée Ligue Nationale Contre le Cancer. 8 quai moncousu, BP7021, 44007 Nantes, France; 2 Université de Nantes, Faculté de Médecine, Département de Recherche en Cancérologie, IFR26, F-4400, Nantes, France; 3 PICell, Plateau technique d'imagerie cellulaire - IFR26 - Centre de Recherche en Cancérologie Nantes-Angers, INSERM, U892. F-4400, Nantes, France

**Keywords:** Global DNA methylation, method, Dnmt1/PCNA, proximity ligation *in situ *technology

## Abstract

**Background:**

DNA methylation has a central role in the epigenetic control of mammalian gene expression, and is required for X inactivation, genomics imprinting and silencing of retrotransposons and repetitive sequences. Thus, several technologies have been developed to measure the degree of DNA methylation.

**Results:**

We here present the development of the detection of protein-protein interactions via the adaptation of the proximity ligation *in situ *technology to evaluate the DNA methylation status in cells since the quantification of Dnmt1/PCNA interaction in cells reflects the degree of DNA methylation.

**Conclusion:**

This method being directly realizable on cells, it appears that it could suggest a wide range of applications in basic research and drug development. More particularly, this method is specially adapted for the investigations realized from a weak quantity of biologic materiel such as stem cells or primary cultured tumor cells for examples.

## Background

DNA methylation has a central role in the epigenetic control of mammalian gene expression, and is required for X inactivation, genomics imprinting and silencing of retrotransposons and repetitive sequences [1,2]. The loss or the decrease of the 5-methylcytosine number on DNA, i.e. the DNA hypomethylation process, is ones of the earlier hallmarks described in neoplasia [3]. Moreover, several paper reports that the DNA hypomethylation is an oncogenic event leading the chromosomal instability and the oncogenes expression [4,5]. Literature also mentioned that the degree of DNA hypomethylation has been also associated with the tumor progression and with the prognosis of survival of patients suffering of tumors [6]. Conjugated with the fact that the DNA methylation abnormalities are potentially reversible, all these points encouraged the development of pharmacologic inhibitors of DNA methylation and their use in anti-tumor therapies. Thus, the quantification of degree of DNA methylation in cells appears as a crucial point for its use in diagnosis, prognosis and the evaluation of the response to treatments including DNA methylation modulators.

Several technologies have been developed to measure the degree of DNA methylation [7]. A first group of technologies is focused around the use of methodologies that can quantify the 5-methyl cytosines using reversed-phase high performance liquid chromatography (RP-HPLC), two dimensional thin layer chromatography (2D-TLC), high performance liquid chromatography-mass spectrometry (HPLC-MS), high performance capillary electrophoresis (HPCE) and liquid chromatography-electrospray ionization-tandem mass spectrometry (LC-ESI-MS/MS) [8,9]. A second group of technologies has been developed to quantify the global 5-methylcytosine via the radiolabelling of CpG sites, ELISA method, pyrosequencing, use of methyl-sensitive enzymes in COBRA method for example [10,11]. Literature also mentions a third group of technologies in which the degree of DNA methylation is analyzed by measuring the activity of enzymes catalyzing the DNA methylation: the DNA methyltransferases [12]. However, all these methods require multiple steps of works and a sufficient (and significant) quantity of cells initially, and this point is not always possible especially by working with certain primary cultured tumor cells. Thus, and in spite of the fact that the method here exposed is an indirect method of quantification of DNA methylation, it provides the possibility to estimate the DNA methylation status from a weak quantity of biologic materiel such as stem cells or primary cultured tumor cells for examples.

## Results

**Figure 1:**
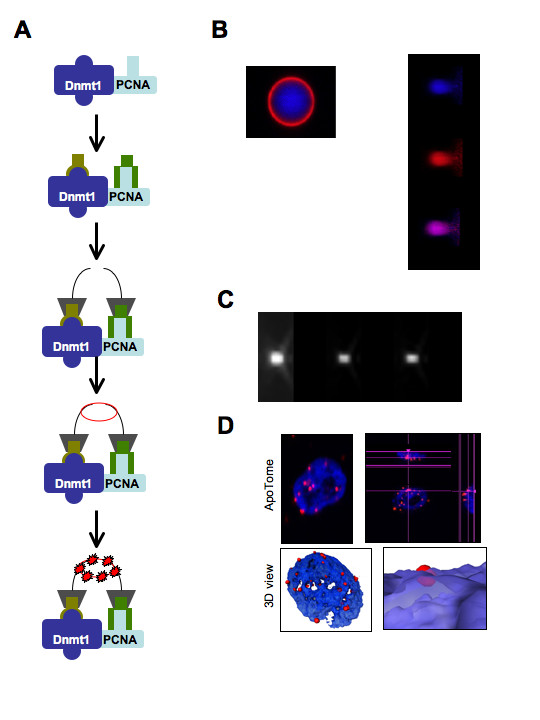
**Detection of endogenous Dnmt1/PCNA interactions using P-LISA**. (A) Schematic representation of the Dnmt1/PCNA proximity ligation *in situ *assay (P-LISA). (B) Calibration of the microscopy (Axiovert 200 M Zeiss, Le Pecq, France) with ApoTome module. Left: picture of calibration performed with calibration balls (4 μm, Vue X-Y, Molecular Probes F36909). Right: picture of calibration performed with calibration balls (2.5 μm, Vue X-Z, Molecular Probes F36909). Bleu and red were merged in lateral and axial. (C) Decovolving of picture realized with calibration balls (140 nm). (D) Dnmt1/PCNA interactions were visualized in MCF10A cells. Red dots symbolize Dnmt1/PCNA interactions. Nucleus/DNA are stained in blue via the use of DAPI. Top left: ApoTome view, top right: orthogonal view, bottom: 3D views.

The technology that we present here is based on the detection of protein-protein interactions via the adaptation of the proximity ligation *in situ *technology to the context of DNA methylation [13]. Based on the demonstration that the interaction between Dnmt1 and PCNA plays a crucial role into the DNA methylation inheritance, we decided to monitor and quantify the Dnmt1/PCNA interaction by proximity ligation *in situ *assay (P-LISA) in cells [14,15]. As illustrated by the Figure 1A, the principle of this assay is based on the staining of Dnmt1 and PCNA proteins by two antibodies, which are next revealed by secondary antibodies conjugated with oligonucleotides. In presence of hybridization solution and ligase, the two oligonucleotides form with PLA a circle in case of close proximity of proteins i.e. Dnmt1 and PCNA, here. Then, polymerase and nucleotides participate to the formation of the rolling circle amplification, which are visualized in red fluorescence. Pictures were realized by using microscopy (Axiovert 200 M Zeiss, Le Pecq, France) with ApoTome module. To take in consideration the possible presence of chromatic aberration, we have performed calibration with specific balls of calibration akin the P-LISA signal (Figure 1B). The signal being inferior to the axial resolution and in order to reduce the diffraction, we next decided to decovolve the image by using the Huygens Essential software (Figure 1C). Next, the Huygens's decovovling is used with the "nipkow disk" method. The decovolving makes it possible to solve a signal of low diameter and to increase the quality of the 3D reconstitution. The 3D reconstitution is obtained by using Amira.4.1.1 program.

By using ApoTome technology, we can visualize the interactions of endogenous proteins Dnmt1 and PCNA (Figure 1D). Moreover, the use of this method validated the idea that the Dnmt1/PCNA interactions occur in nucleus and permitted to quantify the number of interaction per nucleus.

**Figure 2:**
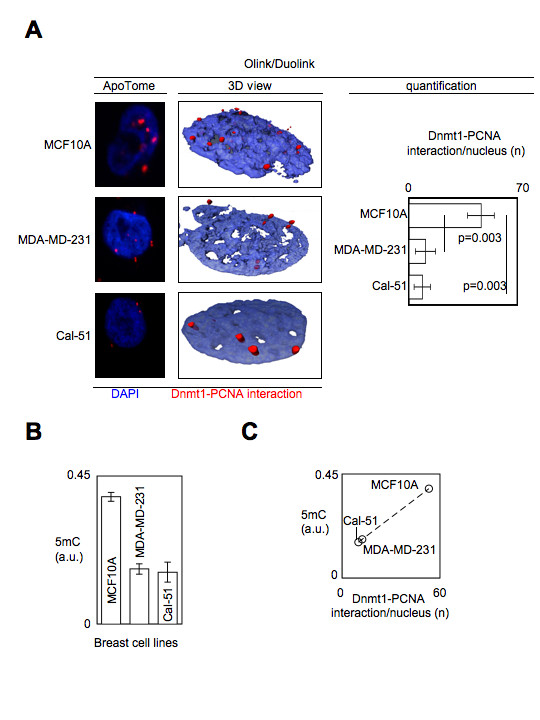
**Decrease of Dnmt1/PCNA interaction is associated with the decrease of the DNA methylation**. (A) Dnmt1/PCNA P-LISA in non-tumor cells (MCF10A) and in tumor cells (MDA-MD-231 and Cal-51). Red dots symbolize Dnmt1/PCNA interactions. Nucleus/DNA are stained in blue via the use of DAPI. The number of Dnmt1/PCNA interactions is calculated from the analysis of 100 nuclei. (B) Evaluation of the degree of DNA methylation in cells by using 5 mC-ELISA. (C) Correlation between the 5 mC (5-methylcyctosine) number and the number of Dnmt1/PCNA interactions.

To validate the possibility to use the Dnmt1/PCNA P-LISA to evaluate the degree of DNA hypomethylation in cells, we compared whether the number of Dnmt1/PCNA interactions was correlated with the values of 5 mC number quantification (ELISA method) in non-tumor and tumor breast cell lines. P-LISA and ELISA experiments realized from non-tumor and tumor breast cell lines revealed that that tumor cells (MDA-MD-231 and Cal-51) harbored less Dnmt1/PCNA interactions and less 5-methycytosine (5 mC) than non-tumor cells (MCF10A) (Figures 2A and 2B). In others terms, these results suggest that the DNA is less methylated in tumor cells than in non-tumor cells since tumors cells harbored less Dnmt1/PCNA interactions. By plotting the value of 5 mC and the number of Dnmt1/PCNA interactions per nucleus against each other, it appears that the level of 5 mC was correlated with the number of Dnmt1/PCNA interactions per nucleus (Figure 2C).

**Figure 3:**
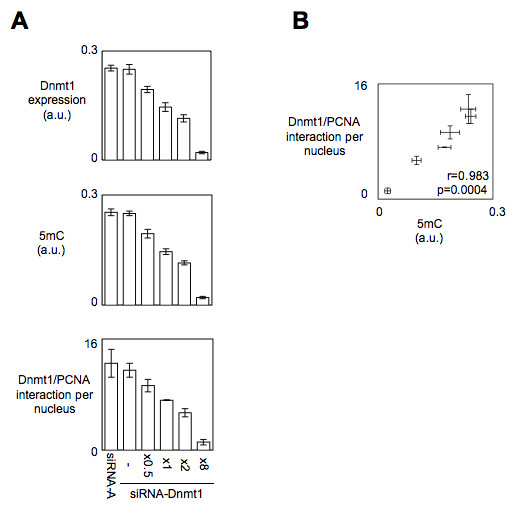
**siRNA-mediated down-expression of Dnmt1 promoted the decrease of Dnmt1/PCNA interactions and the decrease of 5 mC in U251 cells**. (A) Impact of the siRNA-Dnmt1 treatments on the expression of Dnmt1 (ELISA method according to our previous report [17]), on the 5 mC (ELISA method) and on the number of Dnmt1/PCNA interactions (P-LISA method). siRNA-Dnmt1 (sc-35204, Tebu-Bio, France) (B) Correlation between the 5 mC (5-methylcyctosine) number and the number of Dnmt1/PCNA interactions.

**Figure 4:**
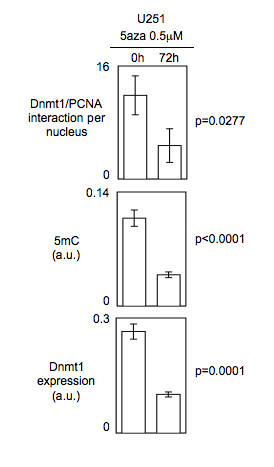
**Impact of the 5aza treatment on the DNA methylation status (ELISA method), the number of Dnmt1/PCNA interactions (P-LISA method) and on the Dnmt1 expression (ELISA method) in U251 cells**.

To test the idea that the Dnmt1/PCNA interaction can be used to estimate the level of global DNA methylation, we analyzed the effect of DNA hypomethylating agents/strategies on the Dnmt1/PCNA interactions. Thus, we firstly monitored the 5 mC number and the Dnmt1/PCNA interactions in cells harboring an increasing deficiency of Dnmt1 expression. For this purpose, we used U251 cells and siRNA directed against Dnmt1. As illustrated by the Figure 3A, ELISA monitoring the Dnmt1 expression indicated that the Dnmt1 expression decreased when the quantity of siRNA treating the cells increased. ELISA assessing the 5 mC and P-LISA measuring the Dnmt1/PCNA interactions revealed that the siRNA-mediated decrease of Dnmt1 expression was correlated with the decrease of 5 mC and with the decrease of Dnmt1/PCNA interactions (Figure 3A). In addition, a Pearson's correlation test also showed a significant correlation between the number of Dnmt1/PCNA interactions and the number of 5 mC (r = 0.983, p = 0.0004) (Figure 3B). Secondly, we analyzed the effect of the 5-aza-2-deoxycytidine treatment on the number of Dnmt1/PCNA interactions and on the DNA methylation status in U251 cells. In absence of toxic effect of our 5aza treatment, we observed, in parallel, significant decreases of the number of Dnmt1/PCNA interactions, of the DNA methylation status and of the level of the Dnmt1 expression (Figure 4). Through these two last experiments, we noted that the decrease of the number of Dnmt1/PCNA interactions is associated with the decrease of 5 mC under a context of decrease of Dnmt1 expression.

**Figure 5:**
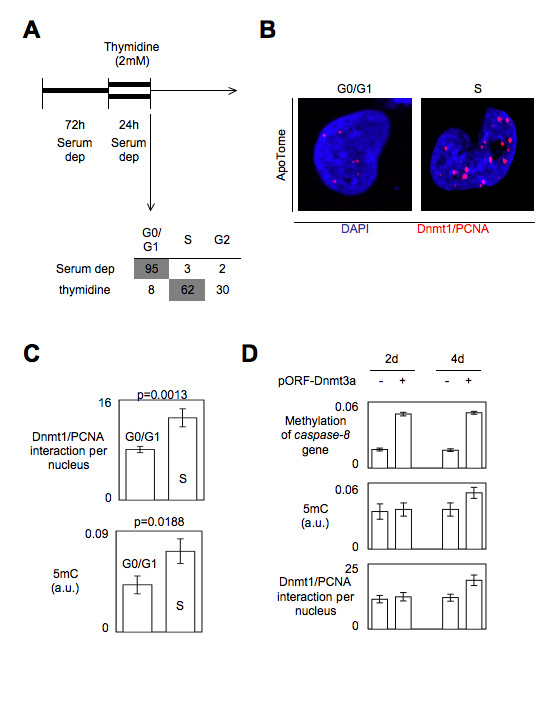
**Impact of conditions increasing the level of DNA methylation on the number of Dnmt1/PCNA interactions**. (A) Characterization cell cycle of arrest. U251 cells were treated by serum deprivation (72 h) or by thymidine (2 mM, 24 h), previous to perform cell cycle analysis by using the NucleoCounter NC-3000™ Kit, Chemometec, France). (B) Dnmt1/PCNA P-LISA in U251 cells blocked in G0/G1 or S phases of the cell cycle. Red dots symbolize Dnmt1/PCNA interactions. Nucleus/DNA are stained in blue via the use of DAPI. (C) Analysis of the number of Dnmt1/PCNA interactions (P-LISA) and the 5 mC number (ELISA). The number of Dnmt1/PCNA interactions is calculated from the analysis of 100 nuclei. (D) Impact of the Dnmt3a overexpression in U87 cells on the level of methylation of the capase-8 gene (MeDIP), on DNA (5 mC ELISA) and on the number of Dnmt1/PCNA interactions. The number of Dnmt1/PCNA interactions is calculated from the analysis of 100 nuclei. U87-pORF-Dnmt3a and the U87-pORF (transfection control (-)) were obtained as previously described [16].

After the analyses of the conditions reducing the Dnmt1/PCNA interactions and/or the degree of DNA methylation, we now considered the condition increasing the Dnmt1/PCNA interactions and/or the degree of DNA methylation. Physiologically, the degree of DNA methylation increases between the G1 and S phases of the cell cycle since, after the DNA replication, the neo-synthesized strand of DNA is methylated according to the pattern of methylation of the parental strand in order to promote the DNA methylation inheritance process. Thus, we next analyzed the evolution of the degree of DNA methylation and of the number of Dnmt1/PCNA interaction when cells were in G1/G0 and S phases of the cell cycle. For this purpose, U251 cells were blocked in G0/G1 and S phases by using serum deprivation and thymidine treatments after a first synchronization by serum deprivation (Figure 5A). P-LISA and 5 mC ELISA were realized in parallel to monitor the number of Dnmt1/PCNA interactions and the degree of DNA methylation. As illustrated by the Figures 5B and 5C, we observed that the significant increase of Dnmt1/PCNA interactions in S phase is associated with the significant increase of the 5 mC number (p = 0.0013 and p = 0.0188).

The degree of DNA methylation can also increase when cells are performing *de novo *methylation by Dnmt3. To aim this situation, we have used the U87-pORF-Dnmt3a cells since we have already used these cells to promote the methylation of the *caspase-8 *gene [16]. Under this condition, we observed that the global level of DNA methylation and the number of Dnmt1/PCNA interactions unchanged 2-days after the cells transfection (p = 0.7262 and p = 0.4918) (despite the fact that the methylation level of caspase8 gene was already increased at this point (p < 0.0001)), while we observed an increase of the global level of DNA methylation and of the number of Dnmt1/PCNA interactions 4-days after the cells transfection (p = 0.0292 and p = 0.0142) (Figure 5D). Interestingly, these data suggest that the number of Dnmt1/PCNA interactions increases in response to the Dnmt3a-induced increase of the 5 mC number. However, the Dnmt1/PCNA interactions monitoring was not representative of the methylation degree of the *caspase-8 *gene. Thus, these data suggest that the Dnmt1/PCNA interactions monitoring is not recommended to analyze the methylation of level of specific gene.

## Discussion

In terms of methodology, all our data argue that the Dnmt1/PCNA Proximity-Ligation *In Situ *Assay presented here should enable to reflect the degree of DNA methylation in cells. Indeed, that its is following a DNA hypomathylation strategy (inhibition of the Dnmt1 expression or treatment (5aza) or by comparing non tumor cells with tumor cells, the number of Dnmt1/PCNA interactions echoes the degree of DNA methylation. In addition this method being performed from fixated cells, it is adapted for investigation realized from weak quantity of biologic materiel (Stem cells, biopsies samples...) on contrary to the majority of technologies aiming at analyzing the degree of DNA methylation (and mentioned into the introduction section). One other advantage of this method is into the fact that it does no require to transform the cells with plasmid or to extract DNA or nuclear samples in order to perform 5 mC-ELISA, to measure of mMTase, for example. In addition, our data indicate that it is not necessary to take into consideration the expression level of Dnmt1 and/or PCNA since none correlation between the Dnmt1 level expression and the 5 mC number was observed in a large number of glioma and 2) since the Dnmt1/PCNA interactions can be inhibited by other events that the level expression of these proteins (such as the phosphorylation of Dnmt1, the presence of peptide/protein imitating the action of the UP peptide [17,18].

Supported by these points, we thus envision a wide range of applications for the Dnmt1/PCNA P-LISA in basic research and into the development of DNA hypomethylation strategies based or not on the inhibition of the complexes including the Dnmt1/PCNA interactions such as the one including the Dnmt1/PCNA/UHRF1 which is considered as the main player of the DNA methylation inheritance.

In terms of biologic results, our data show that the breast cancer cell lines (MDA-MD-231 and Cal-51) harbored less Dnmt1/PCNA interactions and less 5-methycytosine (5 mC) than non-tumor breast cells (MCF10A). Thus, breast cancer appears as the second, after glioma, in which we observed that the DNA hypomethylation characterizing the tumor cells is correlated with the decrease of Dnmt1/PCNA interactions [17,18]. Besides, the fact that DNA of breast cancer cells is hypomethylation in comparison with DNA of breast cells is a well-documented point by the literature [19-21]. The fact that the degree of DNA methylation is correlated with the level of Dnmt1/PCNA interactions makes sense with the fact that the Dnmt1 and PCNA, are two crucial actors, with UHRF1, of the main multiprotein complex, promoting the DNA methylation inheritance in mammalian cells [15,22]. In addition, our data indicate that the Dnmt1/PCNA interactions are increased, such as the DNA methylation level, during the S phase in comparison with the number of Dnmt1/PCNA interactions and the DNA methylation level characterizing the G0/G1 phase. Thus, the use of the Dnmt1/PCNA P-LISA confirms a dogma of epigenetic reporting that the DNA methylation inheritance catalyzed by the complex including the Dnmt1/PCNA interactions is a process mainly realized during the S phase [15,22]. In addition, the use of P-LISA could be interesting to monitor the interactions existing between the Dnmt1 protein (the main enzyme catalyzing the DNA methylation inheritance) and different of its interaction partner during the cell cycle. Besides, these investigations are an ongoing subject in our lab and take place into the debate aiming to characterize the dynamism and the kinetic of the DNA methylation inheritance catalyzed by the Dnmt1 during the cell cycle. Our data also indicate that the monitoring of the Dnmt1/PCNA interactions was not representative of the increase of the methylation degree of the caspase-8 gene following the Dnmt3a overexpression, but that at middle term (4-days) the number of Dnmt1/PCNA interactions reflected the Dnmt3a-induced increase of 5 mC. Thus, our data suggest that the monitoring of the Dnmt1/PCNA interactions is not a method adapted to analyze the degree of methylation of a specific gene but is adapted to monitor the degree of DNA methylation. In addition, the increase of Dnmt1/PCNA interactions in Dnmt3a-induced up-methylated cells was not associated with the Dnmt1 or PCNA overexpression (data not shown). Thus, these data suggest that, after a step of *de novo *methylation catalyzed by the Dnmt3 in "mother cells", "daughter cells" could adapt the number of Dnmt1/PCNA interactions to maintain the DNA methylation pattern realized by the Dnmt3 in "mother cells". Thus, there is an adaptation of the cells according its requirements of DNA methylation inheritance and not according the relative quantity of Dnmt1 and PCNA. This point impacts the methodology of the use of the Dnmt1/PCNA P-LISA for monitoring the global DNA methylation in cells since this suggests that it is not necessary to normalize the counting of P-LISA to the protein level of Dnmt1 and PCNA.

## Conclusion

The detection of the Dnmt1/PCNA interactions via the proximity ligation *in situ *assay to the estimate the degree of DNA methylation in cells appears as an attractive method since 1) it is applicable to a weak quantity of cells, 2) it limits the number of protocol step (no DNA extract, no transfection,...), 3) it reflects the fact that cancer cells are hypomethylated in comparison with no tumor cells, and 4) it echoes the effect of DNA hypomethylating agents/strategies.

## Methods

### Proximity ligation in situ assay

Cells were cultivated for 24 h on cover slip. Cells were then fixed with 4% paraformaldehyde in PBS pH7.4 for 15 min at room temperature. Permeabilization is performed with PBS containing 0.5% Triton 100 × 4 for 20 min at room temperature. Blocking, staining, hybridization, ligation, amplification and detection steps were realized according to manufacturer's instructions (Olink Bioscience). During these steps, all incubations were performed in a humidity chamber. Amplification and detection steps were performed in dark room. Fluorescence was visualized by using the Axiovert 200 M microscopy system (Zeiss, Le Pecq, France) with ApoTome module (X63 and numerial aperture 1.4). Preparations were mounted by using ProLong^® ^Gold antifade reagent with DAPI (InVitrogen, France). Pictures acquisition was realized in structured illumination microscopy [23]. Thus, the lateral resolution (rl) is, according to the Rayleigt criteria: rl = 0.61λ/NA (λ: wavelength; NA: numerical aperture of the objective), and the axial resolution, in ApoTome, is defined by the full-width half maximum (FWHMz)

In this equation, λ is the emission wavelength, n is the index of medium refraction, ν is the frequency and α is the angle of opening of the objective as previously described [24]. After decovolving (3.5 Huygens Essential software (SVI)), 3D view was obtained by using Amira.4.1.1 program. Finally, the image were analyzed by using the freeware "BlobFinder available for download from http://www.cb.uu.se/~amin/BlobFinder. Thus, we obtained either number of signals per nuclei since nuclei can be automatically identified. In other terms, the use of this program participates to the normalization, standardization, reproducibility and to the definition of the cut off signal to accept/quantify or not a dot.

### 5methylcytosine ELISA (5 mC-ELISA) and Methylated DNA ImmunoPrcipitation experiments (MeDIP)

DNA was extracted by using the QiaAmp DNA mini Kit (Qiagen, France), and 5 mC-ELISA was performed by using the Methylamp Global DNA Methylation Quantification kit (Epigentek-Euromedex, France). DNA ImmunoPrecipitation (MeDIP) were performed by using MethylCollector™ kit according to the manufacturer's instructions (Active Motif, France). qPCR were realized to analyze the level of DNA methylation of a considered gene with specific primers (s: CAGGAGGTGGAGGTTGC andas: GAGCCCTAGACCCTCCC) and according our previous report [17].

### Genes invalidation experiments

*Dnmt1 *gene was invalidated by using siRNA approach (sc35204, Tebu-Bio France) according to manufacturer's instructions. siRNA-A was used as control (sc37007, Tebu-Bio France). Classical siRNA treatment (referred as x1) was performed by using 60 pmols of considered siRNA.

## Authors' contributions

EH, PH and PFC carried out the experimental study. FMV and PFC designed the experiments. All authors participated in the analysis of the results, contributed in the preparation of the manuscript and approved the final manuscript.
